# Pediatric pineal region tumors: institutional experience of surgical managements with posterior interhemispheric transtentorial approach

**DOI:** 10.1007/s00381-022-05595-4

**Published:** 2022-07-11

**Authors:** Tadanori Tomita, Tord D. Alden, Arthur J. Dipatri

**Affiliations:** Department of Neurological Surgery, Division of Pediatric Neurosurgery, Ann & Robert H. Lurie Children’s Hospital of Chicago, Northwestern University Feinberg School of Medicine, Chicago, IL USA

**Keywords:** Pineal tumor, Pineoblastoma, Germ cell tumor, Midbrain glioma, Superior medullary velum, Occipital transtentorial resection, Neuroendoscopy, Hydrocephalus headings: pineal region tumors

## Abstract

**Purpose:**

Resecting pineal region tumors in children is often challenging. Several approaches have been proposed and practiced. A personal series of pediatric pineal region tumors resected through craniotomy with posterior interhemispheric occipital transtentorial (OT) approach are reviewed. We present the surgical techniques, pitfalls, and their results.

**Material and methods:**

Eighty patients ranging in age from 3 months to 21 years old, and treated over 3 decades were reviewed. Hydrocephalus caused the main presenting symptoms and was noted in 74 patients. It was treated prior to the craniotomy for tumor resection with endoscopic third ventriculostomy (ETV) in 33, external ventricular drainage in 26, and precraniotomy shunt in 15. Nine patients had ETV together with endoscopic biopsy. All patients had a parieto-occipital craniotomy in a prone position. Through a tentorial section, a gross total resection of the tumor was attempted except for germinomas.

**Results:**

The tumor pathology showed 32 germ cell tumors (GCT), 22 benign astrocytomas, 13 pineal parenchymal tumors, 5 ATRTs, 3 papillary tumors, and 5 others. Of GCTs, 18 were teratomas. The extent of resection consisted of 55 gross total resections, 13 subtotal resections, 10 partial, and 2 biopsies with one postoperative death. Hemiparesis in 2, cerebellar ataxia in another 2, and hemiballismus in 1 were transient and improved over time. One had permanent hemisensory loss and another patient had bilateral oculomotor palsy. Postoperative homonymous hemianopia occurred in 2 patients but subsided over a short period of time. Parinaud’s sign was noted in 24 patients, of which 16 were transient.

**Conclusion:**

The posterior interhemispheric OT approach provides a safe route and comfortable access to the pineal region in children. A great majority of postoperative neurological complications are the results of direct manipulations of the midbrain at tumor resection. Identification and preservation of the tumor-brain interface are of paramount importance. GCTs other than teratomas are treated with neoadjuvant chemotherapy and may eliminate the need for craniotomy. Exophytic midbrain JPAs are amenable to resection.

## Introduction

The pineal region is between the posterior third ventricle and the quadrigeminal cistern, centering the pineal gland. Tumors occurring in this location are either limited within the third ventricle or the quadrigeminal cistern or extend in both directions. In this region, the pineal gland and the surrounding para-pineal structures become the tumor origin. The latter include the tectum and the tegmentum of the midbrain, the posterior commissure, the posterior thalami, the superior medullary velum, the superior cerebellar peduncle, and the superior cerebellar vermis. The splenium of the corpus callosum is present superiorly, and the isthmus of the cingulum and the medial temporo-occipital lobe junction laterally. Pineal region tumors can derive from any of these structures.

The tumors originating from the pineal gland are either germ cell tumors (GCT) or pineal parenchymal tumors (PPT). The 2021 WHO classification of tumors of the CNS includes the following pathological types in the CNS GCTs; germinoma, mature teratoma, immature teratoma, teratoma with somatic-type malignancy, embryonal carcinoma, yolk sac tumor, choriocarcinoma, and mixed germ cell tumors. These GCTs are largely divided into two groups, germinoma and non-germinomatous germ cell tumor (NGGCT). The same 2021 WHO classification includes the “pineal tumor” category, pineocytoma, pineal parenchymal tumor of intermediate differentiation (PPTID), pineoblastoma, papillary tumor of the pineal region, and desmoplastic myxoid tumor (DMT) of the pineal region, SMARCBi-mutated. Papillary tumor of the pineal region is considered to derive from specialized ependymal cells of the subcommisural organ [1]. DMT is a SMARCB1-deficient tumor of the pineal region encountered in adolescents and adults with distinct histopathological features and intermediate prognosis despite epigenetic similarities with ATRT-MYC [2].

Extra-pineal or para-pineal tumors during childhood that derived from the surrounding neural structures are primarily gliomas. They are often exophytic in nature, extending to the pineal region. Glial cell tumors can occur in the pineal gland because astrocytes are normally present there but only compose 5% of the pineal gland cells. Almost all glial tumors arise from the glial tissue elements of para-pineal structures intimately surrounding the pineal gland. The most common histological type is astrocytomas, which often originate in the midbrain or the thalamus. Tumors of mesenchymal origin, such as meningioma, are exceeding uncommon in children.

Pineal region tumors tend to present with obstructive hydrocephalus. Endoscopic third ventriculostomy (ETV) has been a treatment of choice for the treatment of hydrocephalus, together with an endoscopic tumor biopsy. When the endoscopic biopsy is not feasible, a stereotaxic biopsy may be carried out. When positive results of tumor biopsy or tumor markers have led to a diagnosis of GCT, the patients are treated with neoadjuvant chemotherapy. Often this approach adopted by recent multicenter trials alleviates aggressive tumor resection dependent on their responses to the chemotherapy [3–6]. However, they need an open tumor biopsy or resection in the cases of negative tumor markers, or unsuccessful tumor biopsy. Teratomas, pineal parenchymal tumors, papillary tumors of pineal region and benign gliomas, and embryonal tumors such as ATRT and medulloblastomas need to be resected.

Commonly used surgical approaches are the occipital transtentorial (OT) approach, interhemispheric transcallosal approach, and infratentorial supra-cerebellar (ITSC) approaches. Each approach has advantages and disadvantages [7–9]. The approach should be chosen on the basis of anatomical information from neurodiagnostic images along with the surgeon’s familiarity and confidence with the approach. Tumors of the SMV and upper fourth ventricle are readily accessed through the OT approach [10]. Tumor location and extension and the deep venous system and surrounding neural structures need to be correlated with a planned surgical approach. Also, other factors to be considered when selecting appropriate approaches are patients’ age, presence or absence of hydrocephalus, anatomical character based on the MR findings, and purpose of surgery (biopsy vs. total resection).

This is a retrospective review of cases with pediatric pineal and para-pineal tumors treated at a single institution over the past 3 decades since the neuroendoscopy was introduced. The authors surgically treat pineal region tumors predominantly through a posterior interhemispheric OT approach. The surgical techniques and their results were analyzed, and the future direction of pediatric pineal region tumor management will be discussed.

## Patients and methods

During the period of 1990 through 2019, there were 92 patients who underwent craniotomy for pineal region tumor resection at our institution. Approaches for craniotomy and tumor resection employed were either supratentorial craniotomy or posterior fossa craniotomy. The supratentorial craniotomy included an OT approach through the posterior interhemispheric transtentorial route in 80 patients and an interhemispheric transcallosal approach in 3 patients. Among the posterior fossa craniotomies, the ITSC approach was used in 5, and the trans-vermis and/or IVth ventricle approach in 4.

In this communication, we reviewed and analyzed the data of 80 patients who had an OT approach for pineal region tumor resection. The medical records, surgical notes, and all available radiological imaging and histological findings were reviewed. Types of surgical procedures for hydrocephalus management and histological verification, including results of tumor markers and tumor resections, were reviewed with their outcomes. This study was approved by Lurie Children’s Hospital IRB (#2005–12,692).

### Surgical techniques

The parieto-occipital craniotomy for posterior interhemispheric OT approach and surgical resection are outlined below:

#### Ventriculostomy

For the patients with hydrocephalus, EVD is placed preoperatively or at tumor resection. If the patient has an Ommaya reservoir after ETV or VP shunt, their ventriculostomy catheter is also often externalized to better control brain relaxation during the craniotomy.

#### Patient position

The patient is positioned in the prone position. In older patients, a Mayfield three-pin fixation device is used with the head turned approximately 20° in the opposite direction. This head rotation promotes gravity-assisted retraction of the occipital lobe, which falls away from the falx by gravity without forcible brain retraction. The head is in a neutral position without flexion of the neck. For infants and young children with thin skull thickness, the head is placed on a well-padded horseshoe head holder in the neutral position. Most patients have advanced hydrocephalus, which provides access to the ventricle for intraoperative ventriculostomy for CSF drainage to attain brain relaxation.

#### Skin incision

The craniotomy is usually on the right side unless the tumor extension is more toward the left side. Midline incision extended from the inion to the point 4 cm rostrally to the lambda. The lateral incision from the anterior edge of the midline incision extended perpendicular to the sagittal plane and then curvilinearly toward the top of the ipsilateral mastoid, resulting in a hockey-stick incision.

#### Craniotomy

Two burr holes were made with a power drill on the sagittal midline under neuronavigational guidance: one burr at 3–4 cm rostrally to the lambda and another burr hole rostral to the torcular Herophili. The burr hole is widened with a rongeur to expose the dura on both sides of the sagittal sinus. The third burr hole is made 3.5 to 4 cm away from the midline on the lambdoid suture. A parieto-occipital craniotomy crosses the skull midline to the contralateral side by 1–1.5 cm, and the width of craniotomy of the ipsilateral side is 3.5 to 5 cm from the midline.

#### Dural opening/interhemispheric approach

**Fig. 1:**
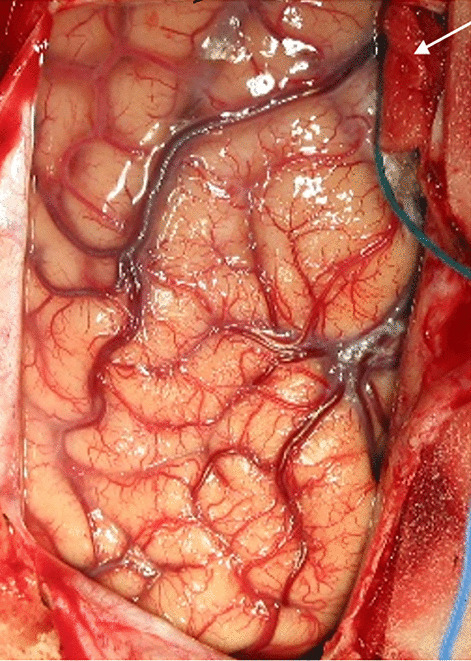
Surgical photograph of exposed left parieto-occipital lobe. The last cortical bridging vein at the entry site to the superior sagittal sinus is protected with a cottonoid pledget (*arrow*)

A surgical microscope and microsurgical instruments are used. The dura is incised and reflected over the exposed superior sagittal sinus. The last posterior cortical vein is identified as running rostrally parallel to and entering the superior sagittal sinus usually 2–3 cm rostral to the lambda (Fig. 1). Upon entering the interhemispheric fissure, which is usually free from any bridging veins, brain relaxation is achieved with drainage of the CSF through the ventriculostomy and by further rotating the operating table in the ipsilateral direction. Brain retraction is applied along the falx at the parieto-occipital junction toward the splenium where the pericallosal cistern is opened to drain the subarachnoid CSF. Then, the quadrigeminal cistern is accessed and opened for further CSF drainage. If occipital lobe prolapse occurs through the craniotomy site or dural opening upon brain retraction, further enlarging of the craniotomy or dural opening avoids brain incarceration. Once the splenium is identified, the straight sinus and the tentorium are visualized posteriorly. The vein of Galen is identified at the posterior inferior edge of the splenium, but the view of the vein may be obscured due to its acute vertical angle.

#### Tentorium section

**Fig. 2:**
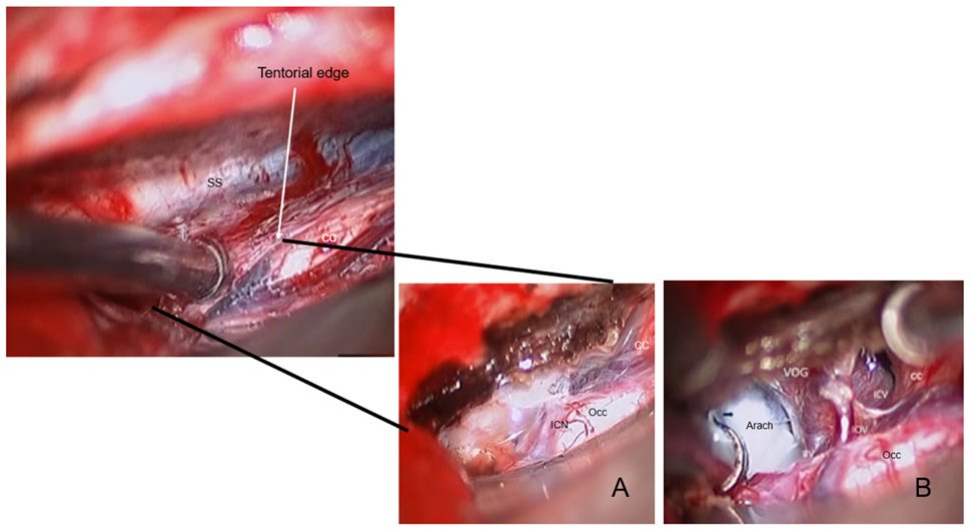
Surgical photo showing the tentorial opening. The tentorium is sectioned (**A**) and the arachnoid membrane is open (**B**), showing the vein of Galen and its tributaries. Note a thick arachnoid membrane (*arach*) over the tectum. (SS: straight sinus, ICN: internal occipital vein, Occ: occipital lobe, cc: corpus callosum, vG: vein of Galen, ICV: internal cerebral vein)

The tentorium is exposed by displacing the occipital lobe laterally and the lateral sinus is identified. The tentorium is coagulated with bipolar cautery about 1 cm laterally to the straight sinus. Venous lakes may be extended from the straight sinus and need to be coagulated thoroughly. The edges of the sectioned tentorium are coagulated, which widens the tentorial opening exposing the underlying cerebellum. The length of the tentorial opening is usually 2 cm, but the tentorial section may extend near its entirety as needed up to the anterior edge of torcular herophili for wider exposure of the posterior fossa structures. Once the tentorium is sectioned and the arachnoid membrane is open, the vein of Galen and its tributary become in the surgical field (Fig. 2). The thick arachnoid membranes covering the quadrigeminal cistern are sharply dissected exposing the deep veins and the tectal plate.

#### Tumor exposure

**Fig. 3:**
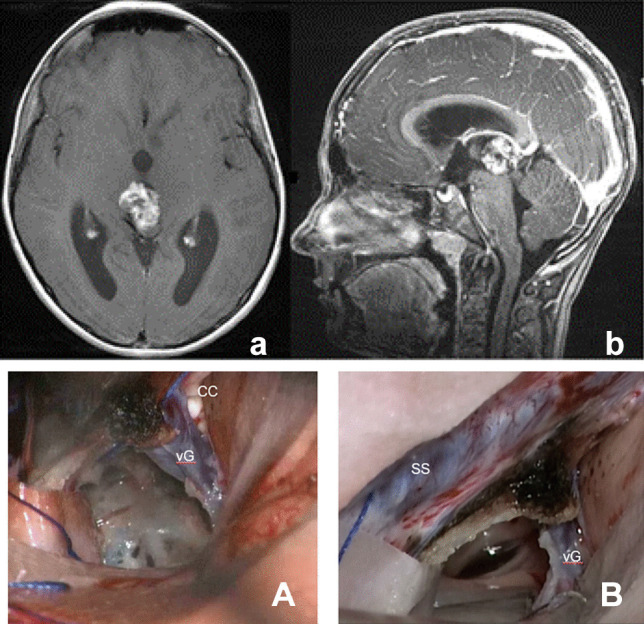
A 10-year-old male with pineal teratoma. Contrast-enhanced MR, axial (**a**), and sagittal (**b**), showing a mass partly protruding to the quadrigeminal cistern. Surgical photo showing the pineal teratoma protruding under the vein of Galen (**A**) and postresection photo through the third ventricle (**B**). (vG: vein of Galen, cc: corpus callosum, SS: straight sinus)

**Fig. 4:**
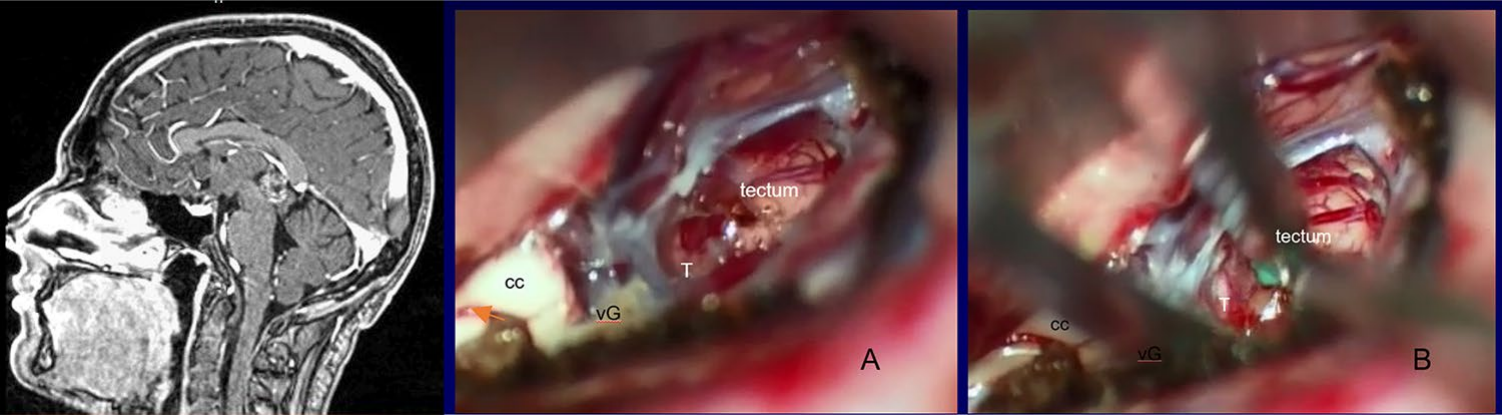
Mid-sagittal postcontrast MR of a 13-year-old male with predominantly intra-third ventricle teratoma. Surgical photo, before (**A**) and after (**B**) exploring the tumor capsule between the vein of Galen and the tectum. (cc: corpus callosum, vG: vein of Galen, T: tumor)

**Fig. 5:**
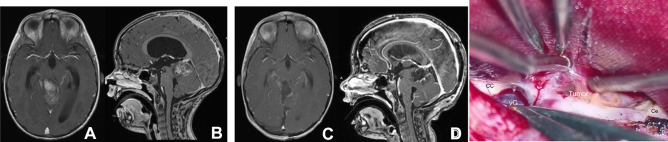
A 5-year-old girl with exophytic tectal JPA. Post-contrast MR images, before (**A** axial, **B** sagittal) and after (**C** axial, **D** sagittal) resection show a heterogeneously enhancing tumor extending to the quadrigeminal cistern. Surgical photo showing a view of an exophytic tumor through the tentorial opening (cc: corpus callosum, vG: vein of Gallen, Ce: cerebellar vermis)

**Fig. 6:**
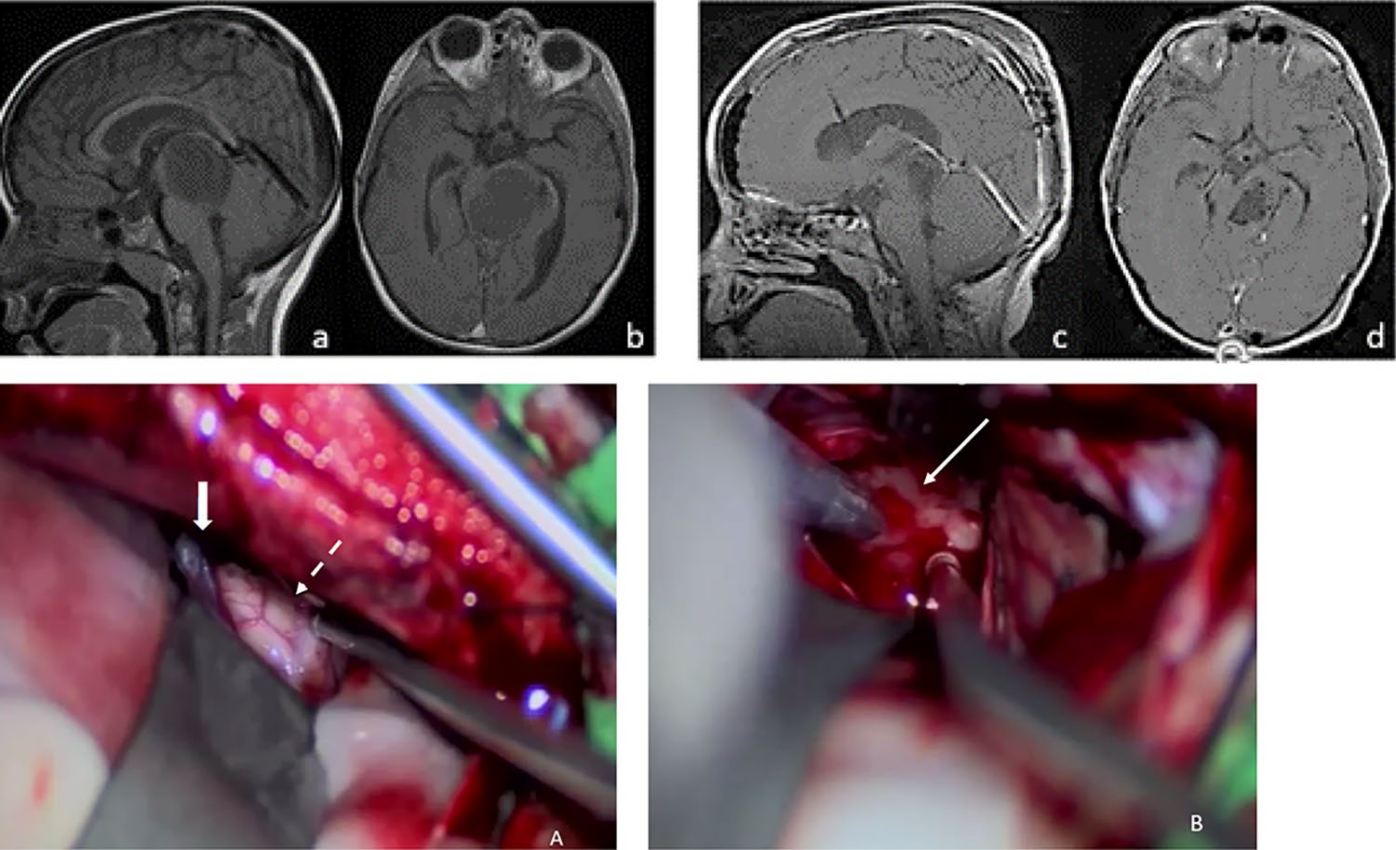
Tegmentum to posterior thalamus JPA. Postcontrast MR before (**a**, **b**) and after (**c**, **d**) tumor resection. Intraoperative photography (**A**) through the left tentorial section shows expanding dorsolateral midbrain (dotted arrow) just next to the vein of Galen (solid arrow). Note tumor-brain interface (arrow) through tumor resected cavity (**B**)

**Fig. 7:**
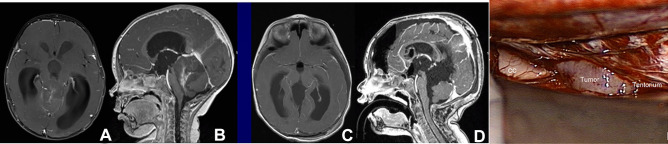
A 13-month-old male with ATRT of the SMV. Post-contrast MR before (**A** axial, **B** sagittal) and after (**C** axial, **D** sagittal) resection through left occipital transtentorial resection. Note the tumor extending to the foramen of Magendie through the IV ventricle. Surgical photo showing a tumor exposed through a tentorial opening (cc: corpus callosum)

Pineal gland tumor is either protruding from the third ventricle to the quadrigeminal cistern (Fig. 3) or maybe within the third ventricle cavity in which the space between the vein of Galen and the tectal plate needs to be created (Fig. 4). Tumors of the midbrain and the thalamus are either exophytic (Fig. 5) or covered by thin neural tissues (Fig. 6). Tumors of the SMV and the superior vermis are present directly underneath the tentorium, which is evident in the tentorial section (Fig. 7). The tumor extends to the posterior fossa, particularly the cerebellomesencephalic fissure (CMF) was approached from a higher angle. If the space between the vein of Galen and the tectum is too narrow to enter the third ventricle, the splenium is sectioned to uncover the underlying tela choroidea and then enter the third ventricle by displacing the internal cerebral veins. After successful resection of the mass from the third ventricle, its anterior wall, including the column of fornices and the foramen of Monro, comes into the surgical view.

#### Tumor resection

Internal debulking is done to bring the outer capsule into the surgical view. For gliomas of the midbrain or the thalamus, the tumor is internally decompressed and then the tumor-brain interface is identified under high-power magnification. Microscope trajectory in AP direction is adjusted depending on the angle to the target; with a more horizontal angle parallel to the skull base at exposing the dome of the tumor in the third ventricle and with a vertical angle at the exposure of the posteroinferior portion, or even to the fourth ventricle. Exposing the contralateral wall of the tumor in the third ventricle is done by adjusting the lateral trajectory toward the end of the tumor resection when the brain is much slack due to loss of the CSF from the third ventricle. The use of an endoscope is helpful for this inspection. An angled endoscope, 30 or 70°, can be used to visualize the contralateral side. In the absence of neuroendocope, a dental mirror is helpful to inspect the contralateral side under the straight sinus.

## Results

Among 80 patients who underwent posterior interhemispheric OT approach for tumor resection, there were 63 males and 17 females, and their ages ranged from 3 months to 21 years, with the mean and median ages being 10.3 years and 10 years, respectively. All but 6 patients had hydrocephalus. All patients were evaluated with MR and head CT.

Primary tumor resection with this approach was performed in 75 patients. The remaining 5 patients had a previous craniotomy for tumor resection. Of these, three were approached through ITSC at outside medical center and had the procedure terminated due to intraoperative hemorrhage. One patient had midbrain tumor resection through a trans-vermis/IV ventricle approach, and another patient had an interhemispheric transcallosal tumor resection, both of which had incomplete tumor resection. Three patients with midbrain benign astrocytoma which failed to chemotherapy underwent subsequent craniotomy for tumor resection.

Prior to or at the craniotomy, hydrocephalus in the 74 patients was managed with ETV in 33 patients, EVD in 26, and existing VP shunt in 15. Of the 33 ETVs, 14 were ETV alone, 9 had additional placement of ETV + EVD at the pre-craniotomy stage, 9 had a concurrent endoscopic tumor biopsy, and 1 had a stereotaxic biopsy for an extra-ventricular pineal region tumor. Another patient without hydrocephalus had a placement of EVD at the time of craniotomy for brain relaxation. The remaining 5 without hydrocephalus had no CSF diversion prior to the tumor resection.

Tumor histology and location are shown in Table 1. There were 32 GCTs, 22 benign gliomas, 13 pineal parenchymal tumors, 5 ATRT, 3 papillary tumor of pineal region, 2 each of medulloblastoma and epidermoid, and 1 thalamic GBM. GCTs were composed of 10 germinomas, 8 teratomas (5 mature and 3 immature), and 14 NGGCTs. Among NGGCTs, 10 patients had mixed GCT composed of teratoma with germinoma and/or yolk sac tumor components. Benign gliomas (18 juvenile pilocytic astrocytoma (JPA), 3 low grade glioma, 1 pilomyxoid astrocytoma) were located in the midbrain tegmentum in 12 patients, the tectum in 3 patients, in the thalamus in 3 patients, in the SMV in 2 patients, and in the superior cerebellar vermis in 2 patients.

**Table 1:** Tumor histology and location

**Tumor origin**	**Histology**	
**Pineal**	**GCT**	**32**
	Germinoma	*10*
	* NGGCT*	*14 (10 with teratoma)*
	* Teratoma*	*8*
	**Pineal tumor**	**16**
	* Pineoblastoma*	*12*
	* Pineocytoma*	*1*
	* Papillary tumor*	*3*
**Para-pineal**		**32**
** Tectum**	* Benign glioma*	*3*
** Tegmentum**	* Benign glioma*	*12*
** Thalamus**	Benign glioma	3
	* Malignant glioma*	1
** SMV**	* ATRT*	5
	* Benign glioma*	2
** Sup. vermis**	* Medulloblastoma*	2
	* Benign glioma*	2
** Cistern**	* Epidermoid*	2

Parieto-occipital craniotomy was performed with posterior interhemispheric OT approach. Three patients had a section of the splenium for additional transcallosal approach. Maximum tumor reduction was intended in all, however in the case of germinomas, the resection was limited intentionally to either biopsy or partial resection once a frozen section disclosed this histology. The extent of the tumor resection was gross total resection in 55, subtotal resection in 13, partial resection in 10, and biopsy in 2.

**Fig. 8:**
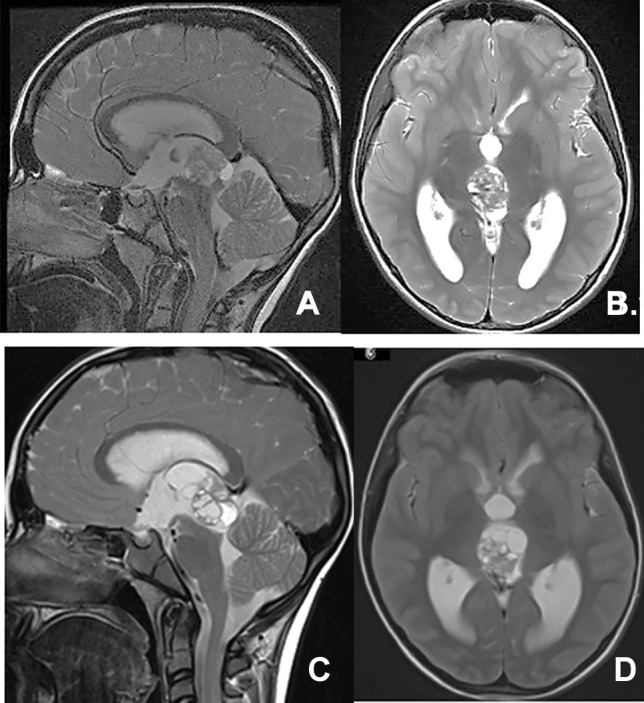
A case of growing teratoma. An 11-year-old boy with an inhomogeneous mass in the posterior third ventricle on T2 weighted MR, sagittal (**A**), and axila (**B**) images. He had elevated AFP (264 ng/mL in serum and 7.0 in the CSF). The following chemotherapy 3 months after neoadjuvant chemotherapy, AFP titer became normalized but the tumor continued to increase on T2 weighted MR, sagittal (**C**), and axila (**D**) images. A mature teratoma was resected on a second-look surgery

All 18 patients with mature or immature teratomas had a gross total resection. Ten of these teratomas were as a component of mixed GCT and were removed at a second look surgery following neoadjuvant chemotherapy for NGGCT. Among them, three had tumor growth during chemotherapy due to a growing teratoma, which was subsequently surgically removed (Fig. 8).

**Fig. 9:**
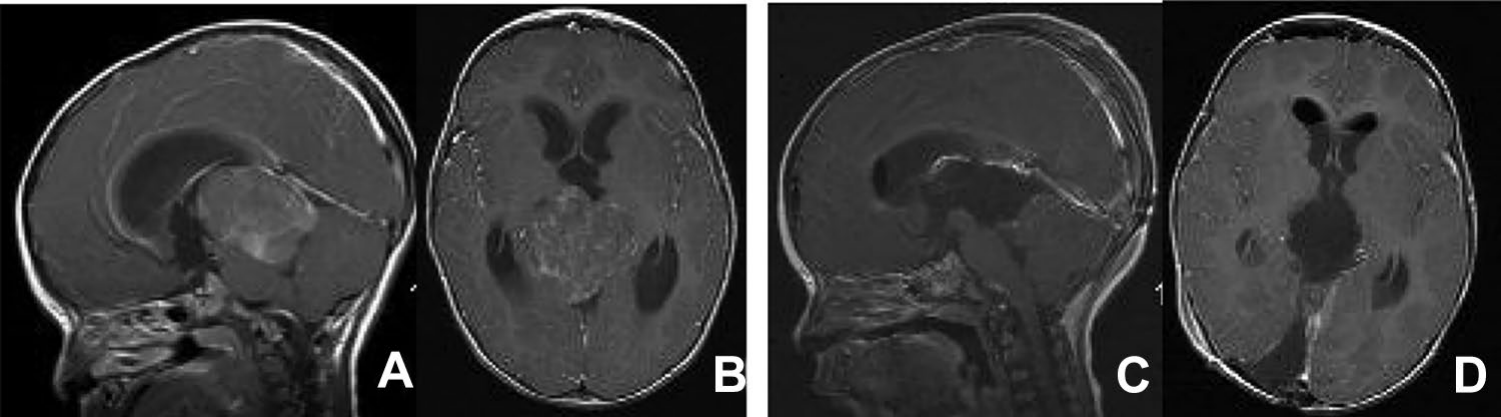
Post-contrast MR, sagittal (**A**), and axial (**B**) images show an enhancing large JPA extending from the posterior third ventricle to the bilateral thalamus. The tegmentum and tectum of the midbrain are obscure. Postresection of JPA, MRI, sagittal (**C**), and axial (**D**) images showing resolution of the tumor mass and re-appearance of the compressed midbrain. The tumor appeared to arise from the right posterior thalamus extending to the third ventricle

Of 12 midbrain tegmentum astrocytomas, 7 had a gross total resection and 3 subtotal and 2 partial resections. Astrocytomas of the tectum were resected in 2 in a gross total fashion, and one with dorsal exophytic extension was partially removed. Three thalamic astrocytomas with an exophytic extension to the posterior third ventricle had gross total resection in 3 (Fig. 9). All JPAs of the SMV and the superior vermis had a gross total resection.

Among pineal tumors, 7 of 12 pineoblastomas, one pineocytoma, and one of the 3 papillary tumors of pineal region had a gross total resection, and the rest were subtotal resections.

All ATRTs and JPAs of the SMV, which is often in a blind spot with the ITSC approach [11], had a gross total resection. Superior vermis tumors, 2 JPAs (and 2 medulloblastomas), and 2 epidermoid tumors had a total resection.

### Post-craniotomy course

Postoperative complications are as follows. There was one postoperative mortality (1.25%). It happened to a 6-year-old boy with an exophytic tegmentum astrocytoma who died of a midbrain dysfunction 6 weeks following partial tumor resection. One patient had a postoperative hematoma in the tumor resected cavity following germinoma resection and needed an urgent reoperation for hematoma evacuation with subsequent uneventful recovery. The progressive subdural collection was noted in 3 infants and needed a subdural peritoneal shunt.

Two patients had postoperative hemiparesis and another two had increased cerebellar ataxia after the removal of the astrocytoma from the tegmentum of the midbrain. All subsequently improved following physiotherapy. Following a resection of JPA of the tectum and tegmentum, junction had hemiballismus, which lasted for a year and subsequently subsided. Another patient with JPA of the tectum partially extending to the tegmentum experienced the postoperative loss of sensation to pain and temperature on the contralateral side of his body and limb, which never recovered over 15 years of the follow-up period.

Common ocular symptoms following surgery were Parinaud signs. It occurred in 24 patients. Of these, 16 were transient and subsided in 6 months to a year. The remaining 8 patients had persistent Parinaud signs, which were only detected by follow up neurological examinations. Parinaud signs occurred more common after resection of pineal GCTs or pineal parenchymal tumors.

Persistent double vison occurred in 6 patients comprising of vertical double vision in 2, horizontal double vision in 2 and exotropia in 2. One teenage boy had bilateral oculomotor nerve palsy following a total resection of papillary tumor of pineal region. Homonymous hemianopia on the contralateral side of the craniotomy occurred in 2 patients, and both were transient and subsided in 2 weeks and 6 weeks, respectively.

### Adjuvant therapy and outcome

All patients with germinoma and malignant NGGCT, diagnosed by either tumor marker or biopsy, were treated with neoadjuvant chemotherapy. Prior to the RT, 7 of these patients had a second look surgery to remove any lesion left following chemotherapy. These patients were enrolled in Children’s Oncology Group (COG) germ cell protocol ACNS0122 and 0232, and most recently in ACNS 1123. All patients with teratoma and epidermoid underwent tumor resection alone. One mature teratoma patient had a recurrence 4 years later when the second surgery disclosed a dermoid. None of patients with pure germinoma had recurrence for over 3 and 30 years. All three patients with immature teratoma following a total resection without adjuvant therapy, had a local recurrent tumor: the first one, a 9-year-old boy, had a primitive neuroectodermal tumor at recurrence a year later. He was treated with chemotherapy and RT. The second one was 21-month-old boy developed a recurrence on follow-up imaging, which was resected and proven to be a mature teratoma. The third one was a 9-year-old boy at diagnosis and developed a recurrence 15 years later which showed a mixed malignant GCT with immature teratoma, germinoma, and yolk sac tumor components. Three patients with malignant NGGCT died despite radio-chemotherapy 6 month, 14 months, and 35 months later due to tumor recurrence.

All but one child with pineoblastoma received chemotherapy followed by RT, including the craniospinal axis. Of 12 patients with pineoblastoma, only 3 are alive between 3 and 8 years. The other 9 patients died of tumor progression between 7 months to 4 years. One patient with pineocytoma who did not receive adjuvant therapy remains alive for 8 years without recurrence.

All 5 infants with ATRT of the SMV, with their ages ranging from 4 to 13 months at the time of diagnosis, received intensive chemotherapy. Of these, only one had subsequent RT. All died of tumor progression with CSF dissemination between 4 and 27 months after diagnosis. Two patients with medulloblastoma received RT followed by chemotherapy without recurrence for 6 to 7 years.

Thirteen patients with totally resected benign astrocytoma were observed without adjuvant therapy except for one child with pilomyxoid astrocytoma who was treated with COG low-grade tumor chemotherapy protocol using vincristine and carboplatin. During the follow-up of 3 to 25 years without adjuvant therapy, only one patient with JPA had recurrence after total resection. Among the remaining patients with incomplete tumor resection, two patients received chemotherapy, and another was treated with laser interstitial thermal therapy for the residual tumor at the outside institution. Two received RT without chemotherapy, one for a recurrent low-grade astrocytoma 3 years after subtotal resection, and another after subtotal resection of a progressive JPA affecting bilateral thalamus and tegmentum.

## Discussion

There was a reduction in the number of patients with germinoma and some with NGGCT who undergo craniotomy for tumor resection because they were treated without craniotomy after the histology is suspected either by biopsy or tumor marker expressions. Thus, the histological distribution of pediatric pineal region tumors presented here does not reflect the true incidence. Schulz reported that histological distribution of pineal region tumors shows GCTs in 36%, pineal parenchymal tumors in 14%, and glial tumors in 46% of the 28 pathologically proven pediatric pineal region tumors [11]. Their histological distribution is similar to our report. Based on a total of 221 lesions in a predominantly adult group, Lin et al. reported pineal parenchymal tumors in 25.3%, glial neoplasms in 18.6%, GCTs in 17.6%, mesenchymal neoplasms in 10.4%, papillary tumors of the pineal region in 6.3%, and glioneuronal tumors in 5.0% [12]. Abecaasis et al. reported 50 pineal region tumors in a mixture of pediatric and adult patients. GCTs in 18 cases were only slightly more common than pineal parenchymal tumors in 13 cases, followed by a papillary tumor in 5 cases and pilocytic astrocytoma in 4 cases [13].

### Historical overviews

Open surgical resection for pineal region tumors has been practiced with several different approaches. In 1921, Dandy initially reported on a parietal interhemispheric transcallosal approach [14]. Cushing in 1932 made the statement that he had “never succeeded in exposing a pineal tumor sufficiently well to justify an attempt to remove it [15]. Dandy in 1936 reviewed his experiences with 10 cases of pineal tumor and reported 7 consecutive deaths during the period and stated that this “seemed almost to indicate the futility of further efforts” [16]. He wrote, “Although an operative approach to the pineal region was proposed by me in 1921, it was not until a decade later (1931) that the first pineal tumor was successfully extirpated” [17]. In the same publication, Dandy reported 3 cases of successful resection of pineal tumors using a posterior interhemispheric transcallosal approach through an occipital craniotomy (Dandy called it “pineal flap”) or a parietooccipital craniotomy with the anterior flap up to the entry of the Rolandic vein. He also described a need for the tentorial section to augment the surgical access to the posterior fossa. For this approach, Dandy used a prone position with the patient’s head secured on a horse shoe head holder [16]. Horrax in 1950 stated that “any operation designed for the removal of a pineal tumor is an extremely hazardous undertaking is well known to all neurosurgeons, even in the present era, when all modern adjuncts can be utilized.” He stressed the importance and far greater safety of the conservative treatment of these tumors by decompression and roentgen therapy rather than by a radical extirpation of the tumor is once more emphasized [18].

Although initial experiences of direct surgical resection were quite discouraging due to high morbidity and mortality rates, the availability of the surgical microscope and more established surgical approaches, advanced neuroimaging, and neuroendoscopic technology have made it possible for neurosurgeons to resect these tumors more effectively with minimal risks. Current practice divides the approaches into supratentorial and infratentorial [19, 20]. The supratentorial route includes an OT approach, posterior interhemispheric approach, posterior interhemispheric transcallosal approach, anterior interhemispheric transcallosal approach (with either subchoroidal route or interforniceal route) and rarely, lateral transventricular approach. The infratentorial approach uses a supra-cerebellar rout through a posterior fossa craniotomy (ITSC approach).

### Infratentorial supra-cerebellar approach

The infratentorial route is primarily approached along the superior surface of the cerebellum and the inferior surface of the tentorium. This infratentorial supracerebellar approach was first described by Krause in 1926 [21] and was refined and popularized by Stein in 1971 [22]. The advantage of the ITSC approach is the midline approach to the midline lesion between the cerebellum and tentorial opening. Usually, it is done with the patient in a sitting position, which allows the cerebellum to fall by gravity and the tumor is readily separated from the vein of Galen. However, this position causes significant discomfort for the operating surgeon through a narrow posterior fossa opening, and his/her arms and hands need to be elevated and outstretched to reach the distant focal target. The superior cerebellar veins, including the precentral vein and superior vermian veins need special attention to assure their preservation [23]. Also, there are concerns about air embolism in the patients. The ITSC approach creates severe limitations accessing the CMF and SMF without forcible retraction or splitting of the superior vermis.

### Interhemispheric transcallosal approach

An anterior interhemispheric transcallosal transchoroid fissure approach may be used through a frontal craniotomy when the third ventricle is totally occupied by a large tumor that further extends into the lateral ventricle either through the foramen of Monro or subcoroidally [24]. In the transchoroidal approach, the third ventricle is exposed by opening the choroidal fissure along the tenia fornicis rather than opening the tenia thalami (the subchoroidal approach), which may risk injury to the thalamostriate vein. Since the tumor mass elevates the roof of the third ventricle in such a case, the subchoroidal space is already widened, and the third ventricular space is readily entered through it. This approach is particularly useful for third ventricle tumors that extend bilaterally into the lateral ventricles.

Transcallosal interforniceal approach through anterior interhemispheric approach has been advocated by others for pineal region tumors [25]. Jia et al. reported a high success rate of gross total resection of the pineal regions in children. However, they had a high incidence of postoperative short-term memory deficit, in 94 out of 150, though the memory deficits are usually transient and resolved within 6 months [25]. This approach, however, carries a potential risk for bilateral damage to the fornices and persistent memory loss.

The posterior transcallosal approach is either through the body of the corpus callosum [26] or through the splenium of the corpus callosum [13]. Often the former approach requires transecting the cortical bridging veins. The preservation of bridging veins during surgery is of importance to decrease the risk of major neurological complications [27]. Dandy was against transecting these bridging veins [17]. Also, tumors in the retrocausal extension or posterior fossa extension are hard to reach by the parietal transcallosal approach. The occipital trans-splenium and transtentorial approach, which was first described by Dandy, causes less chance of sacrificing the bridging veins. It provides better access to the retro-splenial and the posterior fossa through the tentorial section [17]. However, sectioning the splenium of the corpus callosum may cause disconnection syndrome. Resection of tumors of the third ventricle via the interhemispheric transcallosal approach is associated with significant postoperative morbidity by Hassaneen et al. [27].

### Occipital transtentorial approach

Poppen in 1966 [28] developed an OT approach through an occipital craniotomy. This approach was modified by Jamieson in 1971 [29]. Poppen used a lateral occipital craniotomy away from the midline. He approached the pineal region through a suboccipital route by lifting the occipital lobe away from the tentorial surface and then sectioned the tentorium to uncover the subjacent cerebellum (suboccipital approach). Jamieson modified the OT approach and performed a more medial occipital craniotomy. The occipital lobe was displaced laterally away from the falx, and the pineal region was approached through an interhemispheric approach. The tentorium is divided from a point anterior to the transverse sinus forward to its free edge. Glasauer in 1970 [30] exposed both the sagittal sinus and the transverse sinus through an occipital craniotomy and retracted the occipital lobe laterally and upward, exposing the junction of the posterior falx and medial tentorium, then the tentorium was open parallel to the straight sinus (combined approach).

With the suboccipital approach, direct compression of the visual cortex is avoided at occipital lobe retraction. It, however, limits the vertical angle to access the splenium superiorly and the CMF inferiorly. The interhemispheric approach provides a wider exposure of the vertical sagittal angle from the corpus callosum to the upper posterior fossa. It provides upper posterior fossa access to the tumor in the CMF, the SMV, and the upper fourth ventricle ventral to the superior cerebellar vermis [31]. Some may use this approach to access anterior superior vermis lesions [32, 33]. The angles of the microscope trajectory are adjusted according to the target along the sagittal plane.

Disadvantages of the interhemispheric approach are potential concerns of postoperative hemianopia due to retraction of the occipital lobe, diagonal angles to the vein of Galen and its tributaries, and blind spots. Blind spots at the interhemispheric approach are due to limited visibility and access to the contralateral structures of the third ventricle, the tecum, and posterior fossa. Also, lesions deep in the ipsilateral wall require further lateral occipital lobe retraction. The suboccipital approach expands the access to the contralateral side with little lateral or no retraction of the medial occipital lobe [31]. The combined suboccipital and interhemispheric approach allows “a wider angle for maneuverability in the lateral to the medial direction,” which improves the accessibility to the contralateral side [7, 34, 35]. However, it needs further brain relaxation and occipital lobe retraction to expose both the tentorium and the falx wider.

For the OT approach, brain relaxation is essential to gain wider exposure to the pineal region and also avoid neurological complications such as hemianopia. Intraoperative ventriculostomy is the most effective to gain brain relaxation. Additionally, techniques to attain gravity-assisted occipital lobe retraction have been developed, which include the three-quarter prone position [34] or ipsilateral recumbent position [36] with the surgical side down which allows the brain to fall away from the falx by gravity. However, in children with a prone position, it is usually sufficient to turn the head about 15–20° contralaterally. If necessary, the operating table is turned further ipsilaterally to gain more gravity-assisted retraction.

### Complications and their avoidance of interhemispheric OT approach

Our experience using the posterior interhemispheric OT approach to pineal region tumors provides easy access to the pineal region and affords a wider view of the posterior third ventricle and upper posterior fossa structures. The benefit of this approach is a comfort to the surgeon when the patient is placed in a prone position, and usually there is no need to sacrifice the neural and neurovascular structures in order to uncover the tumor. On the other hand, a portion of the tumor across the midline to the contralateral side of the third ventricle or posterior fossa is in a blind spot.

Contralateral homonymous hemianopia is a well-known complication when using the interhemispheric OT approach for pineal region tumors due to occipital lobe retraction. The stria cortex, the primary visual cortex, is located along the superior and inferior sides of the calcarine fissure in the medial occipital lobe. Not only the striate cortex but the extra-striate visual associate cortex also plays a role in visual perception [37]. Thus, forcible retraction upon the medial occipital lobe may cause hemianopia of the contralateral side. Hemianopia is a frequent occurrence postoperatively after the OT approach, but a rapid resolution of the symptom is the rule. Qi et al. reported that 16.1% had postoperative hemianopia following the interhemispheric OT approach in a three-quarter position. The hemianopia was resolved rapidly and only 3.5% had permanent hemianopia [9]. In another series of adult patients reported by Yoshimoto et al. 11 of 14 patients had hemianopia after the interhemispheric OT approach. Their symptoms rapidly diminished and all resolved in less than 3 days [37]. Only 3 patients had prolonged visual field deficits affecting the inferior quadrant noted on the Goldman perimetry test for more than 1 year [37].

The vein of Galen and its ipsilateral tributaries are present dorsally to the tumor in the interhemispheric OT approach. Tributaries include the ICV dorsally, the basal veins of Rosenthal laterally, the internal occipital vein posterolaterally, and the precentral cerebellar and the superior vermian veins at the posterior midline. The vein of Galen and its ipsilateral tributaries are under direct vision. The tumor is approached from the posterolateral angle of the tumor, and these veins are separated from the dome of the tumor. These veins are usually displaced dorsally and distant from the tectal or SMV tumor, but particular attention is needed for tumors of the pineal gland because of their proximity to these veins. The vein of Galen and its tributaries, as well as the pineal body and the supraspinal recess, are typically wrapped by a thickened arachnoid membrane. The internal cerebral veins are within the tela choroidea at the roof of the third ventricle. Preserving their arachnoid covering protects these deep veins. However, there is no intervening arachnoid layer between the pineal gland and the internal cerebral vein. Therefore, tumors arising from the pineal gland are more prone to adhere to the internal cerebral vein, causing a potential risk of venous injury during dissection [38]. The consequences of surgical occlusion of the Galenic system may have been exaggerated [39], but one should try to preserve these deep veins.

Parinaud’s sign is a more common complication. It is well known to be a pathognomonic sign of pineal tumor. Qi et al. reported nearly 40% of patients with NGGCT had it at presentation prior to the surgery [9]. Postoperative Parinaud’s sign is due to direct manipulation of the tectum and posterior commissure, not because of approach. In our series, Parinaud’s sign developed predominantly in patients with pineal GCTs or pineal parenchymal tumors, particularly after radical resection. However, Parinaud’s sign often improves over time, and are often asymptomatic even noticed at follow-up clinical examination.

JPAs of the tectum, the tegmentum, and/or the posterior thalamus are amenable to a resection when there is exophytic extension into the quadrigeminal cistern with or without pia or thin cortical tissue covering. These JPAs can be traced because they tend to be soft and grayish, distinct from the surrounding brain tissue. The safe zones for surgical approach to the midbrain lesion have been described. They include a direct transcollicular approach [40], a peri-collicular route through a transverse incision either just above the superior colliculi or between the inferior colliculi [41], or through the lateral mesencephalic sulcus [42]. However, anatomical surface landmarks are often obscured t due to the underlying mass in the midbrain. If anatomical landmarks are not recognizable, going through the thinnest covering over the tumor is the optimal entry site to uncover the subjacent tumor. Once the underlying tumor is identified, it is removed in a piecemeal fashion. Most JPA is soft and grayish, distinct from the surrounding neural tissues.

However, it is of paramount importance to identify the tumor-brain interface and never go beyond it at tumor resection. Our only postoperative mortality was likely due to a violation of this interface. Hemiparesis, hemianesthesia, cerebellar ataxia, involuntary movement disorder, or oculomotor palsy may occur following aggressive tumor resection from the midbrain. These neurological complications are often temporary and improve over time, but a few may result in permanent deficits, as we experienced.

### Future direction

Neuro-endoscopy has been utilized for pineal region tumor resection over the last decade and is likely to be utilized more in the future. The currently available endoscope aids in uncovering blind spots because of its wider angle of view and its ability to look around corners of the microsurgical field. Endoscope complements the surgical microscope in the detection and removal of residual tumors. Some apply endoscope-assisted microneurosurgery through the ITSC approach with the patient in a sitting position [43, 44] or in a concord position [45]. Through a posterior fossa craniotomy and using the ITSC approach, they felt no fatigue, which is common with microsurgery alone. Others reported endoscope-assisted microneurosurgery through the OT approach. Broggi et al. used an endoscope-assisted interhemispheric OT approach for the excision of 15 pineocytomas. The endoscope enabled the detection of residual tumors located either behind the vein of Galen or attached to the undersurface of the corpus callosum in six cases and guided the residual tumor resection [46]. Shirane et al. reported endoscope-assisted microsurgery through an OT approach with the patient in a Concorde position. The pineal region was approached through a small craniotomy by lifting the occipital lobe superolateral and attained nearly an 80% total resection rate [47].

Fully endoscopic resection has been reported. Sahinian and Ra conducted a fully endoscopic resection of a pineal (posterior third ventricle) tumor through a key-hole craniotomy and ITSC approach [48]. This technique, without any cerebellar retraction or manipulation of neural tissue with a patient in a sitting position, enabled a gross total resection of the pineal region tumor [49]. With endoscopy, Gu et al. could remove pineal region tumors via the space beside the CMF vein without sacrificing it through the ITSC approach [50].

Tanikawa et al. reported the feasibility and effectiveness of the endoscopic OT interhemispheric approach for lesions in these areas, using low or high keyhole craniotomy dependent on the tumor location in the pineal region [51, 52]. They stated that the advantages include minimal invasiveness with a small interhemispheric entrance and limited retraction of the occipital lobe, the elimination of blind spots, and the facilitation of fine manipulation due to the bright, magnified panoramic view. They endoscopically approached tumors extending from the pineal region to the third ventricle by using a low positioned key-hole craniotomy, while lesions around the posterior tentorial incisura and the CMF were approached through a high keyhole.

The advantage of endoscopy is minimum neural retraction of the occipital lobe during the OT approach and the cerebellum during the ITSC approach. The blind spots can be explored using the angled scope. The endoscope provides operating surgeons high-resolution images and more comfortable positioning. The disadvantages of endoscopy should be emphasized as well. The lens itself occupies the operating space and creates a blind area. The learning curve for surgeons may be steep. Adequate practice and training in the laboratory and dexterity should be achieved before performing pure endoscopic surgery [50].

## Conclusion

The posterior interhemispheric OT approach provides safe and comfortable access to the pineal region in children [53]. Occipital lobe retraction needs to be minimized with intraoperative ventriculostomy and gravity assistance. The tentorial section is performed with impunity and provides access to the upper posterior fossa structures. Handling of the vein of Galen and its tributaries needs extra attention. A great majority of postoperative neurological complications are the results of direct manipulation of the tectum and/or tegmentum of the midbrain. Internal decompression enables the identification and separation of the tumor capsule away from the neural tissue for teratomas. For gliomas of the midbrain/posterior thalamus, it needs to be approached along with the exophytic component or through the thinnest covering. Identification and preservation of the tumor-brain interface must be meticulously undertaken following internal debulking of the intrinsic tumor.

Continued advanced imaging and tumor markers would improve the diagnosis of germinoma and NGGCT which often respond to neoadjuvant chemotherapy and decrease the need for craniotomy for these patients. Also, the chemotherapy would reduce the volume and vascularity of the tumor and make subsequent surgical resection safer and more effective. Exophytic midbrain JPAs are amenable to surgical resection albeit with postoperative neurological risks in some patients, while predominantly intrinsic gliomas may be treated upfront with chemotherapy or molecular target therapy.

## Data Availability

Available upon relevant requests.
